# Prevalence, Risk factors, and Outcome of Invasive Fungal Infections in Hematopoietic Stem Cell Transplant Recipients: a prospective cross-sectional study from Iran

**DOI:** 10.22034/cmm.2026.345450.1690

**Published:** 2026-02-01

**Authors:** Nastaran Keyhanian, Mohammadreza Salehi, Seyed Ali Dehghan Manshadi, Seied Asadollah Mousavi, Mohammad Vaezi, Mohsen Meidani, Mohammad Solduzian, Marzieh Shahrabi, Kazem Ahmadikia, Sahar Tavakoli Shiraji, Murat Akova, Johan Maertenes, Neda Alijani

**Affiliations:** 1 Department of Infectious Diseases, Shahid Rajaei Hospital, Mazandaran University of Medical Sciences, Tonekabon, Iran; 2 Research Center for Antibiotic Stewardship and Antimicrobial Resistance, Department of Infectious Diseases, Imam Khomeini Hospital Complex, School of Medicine, Tehran University of Medical Sciences, Tehran, Iran; 3 Cell Therapy and Hematopoietic Stem Cell Transplantation Research Center, School of Medicine, Tehran University of Medical Sciences, Tehran, Iran; 4 Research Institute for Oncology, Hematology and Cell Therapy, School of Medicine, Tehran University of Medical Sciences (TUMS), Tehran, Iran; 5 Hematology, Oncology and Stem Cell Transplantation Research Center, School of Medicine, Tehran University of Medical Sciences (TUMS), Tehran, Iran; 6 Shahid Akbarabad Clinical Research Development Unit (SHACRDU), School of Medicine, Iran University of Medical Sciences (IUMS), Tehran, Iran; 7 Department of Clinical Pharmacy, School of Pharmacy, Tehran University of Medical Sciences, Tehran, Iran; 8 Department of Medical Parasitology and Mycology, School of Public Health, Tehran University of Medical Sciences, Tehran, Iran; 9 Department of Infectious Diseases and Clinical Microbiology, Hacettepe University Medical School, Ankara, Turkey; 10 Department of Microbiology, Immunology, and Transplantation, K.U. Leuven, Leuven, Belgium; 11 Department of Hematology, U.Z. Leuven, Leuven, Belgium; 12 Department of Infectious Diseases, Dr. Shariati Hospital, School of Medicine, Tehran University of Medical Sciences (TUMS), Tehran, Iran

**Keywords:** Aspergillosis, Invasive fungal infections, CMV reactivation, Hematopoietic Stem Cell Transplant, Neutropenia

## Abstract

**Background and Purpose:**

Invasive fungal infections (IFIs) are the second leading cause of infection in hematopoietic stem cell transplant (HSCT) recipients. Although Knowledge of the local epidemiology of breakthrough IFIs should be considered in terms of guiding organizational effective antifungal prophylaxis strategy, however, institutional approach for ongoing surveillance of the rates and epidemiology of IFIs in high-risk patients like HSCT has been less appreciated. Therefore, this study was aimed at investigating the prevalence, risk factors, and outcome of IFIs in HSCT patients.

**Materials and Methods:**

A prospective study was carried out between March 2021 and March 2022 in HSCT wards of an educational – medical hospital of Tehran University of Medical Sciences to rate the incidence of IFIs. All HSCT recipients were enrolled except those who had incomplete files, had a history of previous corticosteroids receipt for rheumatologic or immunologic disorders, or did not consent. Upon suspicion of IFIs, the appropriate sample was collected for mycological assay. Those with proven/ probable IFIs according to the consensus criteria defined by EORTC/MSG were only counted for evaluation of the prevalence, risk factor and outcome of the IFIs.

**Results:**

A total of 330 HSCT recipients were enrolled. The Prevalence of IFIs was 9.6 %, with aspergillosis as the most frequent one being observed in 97 % of the cases. Pulmonary involvement was the most frequent clinical presentation (78.1 %). Most of IFIs were developed before engraftment (90.6%). CMV infection and acute myeloid leukemia (AML) significantly raised the odds ratio of IFIs development (odds ratios of 1.07 and 12.6; p-values of 0.002 and < 0.001, respectively). The mortality rate was 12.5 %. CMV infection, lower age and HLA-mismatch related donor were factors significantly associated with fatality of IFIs (odds ratios of 15.5, 1.07 and 10.70; p-values of 0.005, 0.002 and 0.016 respectively).

**Conclusion:**

IFIs were found to be relatively common complication of allogenic HSCT and continue to be associated with poor prognosis. CMV infection can be independently associated with IFIs and unfavorable outcomes, while HSCT HLA mismatch donors increase the risk of unfavorable outcomes. As a result, tailoring antifungal prophylaxis in these groups of high-risk hosts is crucial.

## Introduction

Infectious diseases in hematopoietic stem cell transplant (HSCT) recipients are serious complications which are associated with significant morbidity and mortality. Invasive fungal infections (IFIs) are the second leading cause of infection in this population of patients with mortality rates of 60–80% for mould infections. Outcomes could be improved by earlier diagnosis, changes in transplant practices, targeted antifungal treatment and less toxic treatment options. Nonetheless, the similar clinical and radiographical patterns, lack of reliable diagnostic tools and the limited number of available treatment options have resulted in a dismal outcome of these infections, especially in developing countries [1].

Published reports indicate that invasive aspergillosis (IA) is the most frequent IFI in these patients, with invasive candidiasis and mucormycosis ranked as the second and third etiology of IFIs [2]. The overall incidence of proven or probable fungal infection in HSCT recipients is about 8 %, with significantly higher rates in allogeneic vs autologous HSCT patients (9.2 % vs 3.5%) [3]. Other studies on these patients have reported overall rates of invasive mold, aspergillosis, and candidiasis to be 8.5%, 6% and 2.3 % respectively. Likewise, 12-week mortality has been reported 20% and 58.6 % in patients with invasive candidiasis or mold infections, respectively [4]. Recent reviews of published studies have reported overall incidence rates of 4% for IFI and 13% mortality rates for patients with these infections [5].

Primary or secondary prevention should be implemented in patients undergoing HSCT in order to lower the risk of acquiring IFIs or to prevent their recurrence. It has been shown that active mold agents might reduce mortality compared to active *Candida* agents and the use of these agents is generally favored, especially in centers with greater prevalence of these types of IFIs [6, 7]. Secondary prophylaxis might be helpful in patients with a history of prior mold infection, especially aspergillosis. Options for the treatment of IFI are limited. Treatment of such infections could be done with liposomal amphotericin B, triazoles and/or echinocandins where appropriate [8]. Prophylactic measures do not always protect against IFIs and current reports show that about 17% of HSCT recipients could develop IFIs in one year following the procedure [9].

Epidemiological studies report a shift on the pattern and distribution of IFI in recent years. The causative agent of invasive candidiasis (IC) cases are shifted from drug-susceptible *Candida albicans* to non-*albicans Candida* (NAC), azole resistant *Candida* species, and molds, with most notably being observed due to *Aspergillus*. The Incidence of mucormycosis infections have also been increasing [10].

In onco/hematology settings, acute myelogenous leukemia (AML), history of multiple chemotherapy sessions, and chronic neutropenia constitutes the major factors predisposing the individuals for IFI development. However, other risk factors might also play a role in a selected population of immunocompromised patients [11, 12]. In HSCT recipients, the HSCT type (allogenic VS autologous) and post HSCT complications (e.g. graft versus host disease (GVHD)) are associated with enhanced risk of IFI development [13, 14]. Other risk factors which could increase the odds of IFI development in HSCT patients include HLA-mismatch related donor, cytomegalovirus (CMV) reactivation, and treatment with corticosteroids for GVHD [15].

Although Knowledge of the local epidemiology of breakthrough IFIs should be considered for guiding effective antifungal prophylaxis strategies and empirical treatment, However, local institutional approach for ongoing surveillance of the rates and epidemiology of IFIs in high-risk patients like HSCT has been less appreciated. The main goal of the current study was to investigate the prevalence, risk factors, and outcome of IFIs in HSCT patients.

## Materials and Methods

The study was conducted as a prospective cross-sectional study during a 12-month period from March 2021 through March 2022 in HSCT wards of Shariati Hospital, affiliated to Tehran University of Medical Sciences (TUMS). All patients who underwent HSCT were enrolled and investigated for the occurrence of IFIs. The study protocol was approved by the local ethical committee. A written informed consent was obtained from each subject or his/her guardians prior to enrolment in the study. Patients who were admitted for a solid organ transplant, those with a history of previous treatment with corticosteroids for rheumatologic disorders or immune dysfunction, and patients who did not consent to participate in the study were excluded.

Proven and probable cases of IFI were defined using the consensus criteria of the Invasive Fungal Infections Cooperative Group of the European Organization for Research and Treatment of Cancer and the Mycoses Study Group of the National Institute of Allergy and Infectious Diseases (EORTC-MSG). Accordingly, for HSCT recipients displaying clinical symptoms consistent with IFI, along with histopathological or microbiological evidence obtained from sterile specimens or biopsied tissues, the condition was considered "proven" IFI. The infection was deemed "probable", If cases exhibited positive direct examination or cultures from non-sterile specimens or elevated GM, as well as IFI associated clinical and radiological signs. those with only ‘possible’ IFI according to the EORTC-MSG consensus criteria were not included in this investigation [16, 17].

Patient’s age, gender, past medical history, type of HSCT, HLA matching status, GVHD prophylaxis regimen, past medication history and medication they received during conditioning for HSCT, central venous catheter (CVC) and its duration and laboratory data including serum and BAL galactomannan (GM) and blood cultures were recorded in patient specific forms. CMV status of the patients was evaluated before admission for HSCT, and those with positive serologic tests were followed preemptively. Patients who were found to be CMV positive by polymerase chain reaction test, were treated using ganciclovir.

Prophylaxis against GVHD was provided using methotrexate and cyclosporine. Regarding reducing the chance of infection occurrence, all patients were receiving antibacterial prophylaxis using fluoroquinolone; against fungal infections fluconazole, itraconazole or voriconazole prophylaxis, and in case of seropositive status, acyclovir against herpes simplex or varicella zoster virus, oral were administrated.

Engraftment was defined as the time when the absolute neutrophil count in peripheral blood sample of the recipient reached 1000 cells/mm^3^ or remained higher than 500 cells/mm^3^ on blood tests of the first three consecutive days.

Accordingly, the occurrence of IFI was characterized in different phases of HSCT process including pre-engraftment (within the first 35-42 days post-transplant), early post-engraftment (within the first 100 days post-transplant) and late post-engraftment (after the first 100 days post-transplant) phase.

Death or prolonged need for antifungal therapy due to IFI during the 3-month follow up period were considered as the primary outcome of the study in cases with IFI. All patients were followed up in the hospital and at the outpatient clinic, every 45 days for 100 days following IFI diagnosis and outcomes of the fungal infections were recorded.

### Ethical Statement

The ethical committee of TUMS reviewed and approved the protocol of study and approval code of IR.TUMS.MEDICINE.REC.1399.710 was dedicated to the study.

### Mycological assays

Direct examinations using 15% KOH and fungal cultures on tissue, BAL and blood specimens were processed at Department of Medical Parasitology and Mycology, School of Public Health, Tehran University of Medical Sciences (TUMS), Tehran, Iran. Isolates were identified to the genus level using morphological appearances.

### Statistical analysis

Statistical analysis was carried out using SPSS software (version 24, IBM Corp, Armonk, New York, USA) and Stata software (Version 14, Stata Corp, College Station, Texas, USA). *P*-value of less than 0.05 were considered statistically significant. Univariate logistic regression analysis was performed to assess the association between fungal infection (dependent variable) and demographics and clinical features (explanatory variables). Any variable which had a significant association at the level of 0.2 was selected for the multiple logistic regression model to assess the simultaneous association of each variable adjusted for the others by the backward stepwise regression approach. These analyses were performed for outcome of fungal infection as dependent variable and other explanatory variables. In order to report data with normal distribution mean ±SD is shown, for data without normal distribution median and interquartile range (IQR), and qualitative data is reported as frequency (%).

## Results

During the study period, a total of 545 recipients were evaluated and finally 330 HSCT recipients were enrolled in the study. Demographic and clinical data of the patients are shown in Table 1 and Table 2. The Mean age of the patients was 37.5 years and 64.5 % of them were men. Overall, 32 (9.6%) cases of IFI were diagnosed after HSCT with only 6 (18.8%) cases being categorized as proven IFI and the remainder were probable IFI. Most of IFIs were developed before engraftment (90.6%).

**Table 1:** Demographic and clinical data of all HSCT patients evaluated.

Data		Report
**Total number of patients evaluated**		330
Age (mean ± SD)		37.5 ± 18.81
Gender	Male N (%)	213 (64.5)
	Female N (%)	117 (35.5)
IFI type	Total N (%)	32 (100)
	Proven N (%)	6 (18.8)
	Probable N (%)	26 (81.2)
Organism detected as the cause of IFI	*Aspergillus* N (%)	31 (96.9)
	*Candida* N (%)	1 (3.1)
Time of IFI development	Pre engraftment N (%)	29 (90.6)
	Early post engraftment N (%)	3 (9.4)
Site of infection	Pulmonary N (%)	25 (78.1)
	Sinus N (%)	12 (37.5)
	Blood N (%)	1 (3.1)
	Other^1^ N (%)	3 (9.4)
Hematologic malignancy	Acute lymphoblastic leukemia N (%)	58 (17.6)
	Acute myelogenous leukemia N (%)	50 (15.2)
	Hodgkin lymphoma N (%)	38 (11.5)
	Myelodysplastic disorder N (%)	8 (2.4)
	Multiple myeloma N (%)	106 (32.1)
	Other^2^ N (%)	44 (13.3)
Type of HSCT	Allogenic N (%)	156 (47.3)
	Autologous N (%)	174 (52.7)
HLA	Mismatched N (%)	46 (29.5)
	Matched N (%)	110 (70.5)
Donor type	Related N (%)	137 (87.8)
	Unrelated N (%)	19 (12.2)
GVHD prophylaxis regimen in allogenic HSCT recipients	Methotrexate and cyclosporine N (%)	156 (100)
GVHD N (%)		50 (15.2)
Conditioning regimen	Myeloablative N (%)	319 (96.7)
	Non-Myeloablative N (%)	10 (3)
	Monoclonal antibody N (%)	1 (0.3)
Type of antifungal prophylaxis	Fluconazole N (%)	265 (80.3)
	Itraconazole N (%)	39 (11.8)
	Voriconazole N (%)	26 (7.9)
History of fungal infections N (%)		33 (10.0)
History of CMV infection N (%)		32 (9.7)
Duration of central venous catheter use (days) (mean ± SD)		25.48 ± 9.34
Duration of neutropenia following HSCT	Longer than 10 days N (%)	140 (42.4)
	Less than 10 days N (%)	190 (57.6)
Central venous catheter N (%)		330 (100)
History of IV drug abuse N (%)		5 (1.5)
Recent surgery N (%)		5 (1.5)
Major comorbidities	Diabetes N (%)	20 (6.1)
	COPD N (%)	1 (0.3)
Mucositis N (%)		209 (63.5)
Laboratory findings indicating fungal infection	Positive galactomannan N (%) (indicating *Aspergillus*)	3 (0.9%)
	Positive blood culture N (%) (indicating *Candida*)	1 (0.3%)
Final outcome	Improvement N (%)	15 (46.9)
	Ongoing therapy following discharge N (%)	13 (40.6)
	Death N (%)	4 (12.5)

IFI, Invasive Fungal Infection; HSCT, hematopoietic stem cell transplantation; HLA, human leukocyte antigen; GVHD, graft versus host disease; CMV, cytomegalovirus; COPD, chronic obstructive pulmonary disease.

1 other sites of IFI infections included skin in 1 case, disseminated in 1 case, and gastrointestinal in 1 case.

2 other diagnosis included aplastic anemia in 18 patients, thalassemia major in 11 patients, Wilms tumor in 3 patients, severe combined immunodeficiency in 4 patients, primitive neuro-ectodermal tumors (PNET) in 3 patients, hemophagocytic lymphohistiocytosis in 3 patients, and mucopolysaccharidosis in 2 patients.

**Table 2:** Characteristics of 32 patients with invasive fungal infection (IFI).

Characteristics		All patients (%)	HSCT type			
			Allogenic (n=21)		Autologous (n=11)	
			IFI cause		IFI cause	
			Mold	Yeast	Mold	Yeast
	No. (%) of patients	32 (100)	21(65.62%)	-	9(28.12%)	2(6.25%)
Age, median years (range)	Ratio of male to female patients	1.9:1	14/7	-	6/3	1/1
Underlying hematological disease	AML	7(21.87)	7(21.87)	-	-	-
	ALL	9(28.12)	9(28.12)	-	-	-
	CML	-	-	-	-	-
	CLL	-	-	-	-	-
	Non-Hodgkin lymphoma	1(3.12)	-	-	1(3.12)	-
	Hodgkin disease	6(18.75)	-	-	6(18.75)	-
	MM	2(6.25)	-	-	1(3.12)	1(3.12)
	Others	7(21.87)	5(15.62)		1(3.12)	1(3.12)
Donor	Related	16(76.19)	16(76.19)	-	-	-
	Unrelated	5(15.62)	5(23.81)	-	-	-
HLA Type	Match	10(41.7)	10(41.7)	-	-	-
	Mismatch	11(52.38)	11(52.38)	-	-	-
Engraftment	Pre engraftment	29		-		
	Early post-engraftment	3		-		
GVHD	Yes	9(28.12)	9(28.12)	-	-	-
	No	23(71.87)	12(37.5)	-	9(28.12)	2(6.25)
Site of IFI	Lung	15(46.75)	8(25)	-	7(21.87)	-
	Sinopulmonary	10(31.25)	9(28.12)	-	1(3.12)	-
	Sinus	2(6.25)	1(3.12)	-	1(3.12)	-
	Blood	2(6.25)	-	-	-	2(6.25)
	CNS		-	-	-	-
	Disseminated	3(9.37)	3(9.37)	-	-	-
CMV serologic test result	Negative	22(68.75)	12(37.5)	-	8(25)	2(6.25)
	Positive	10(31.25)	9(28.12)	-	1(3.12)	-
CVC in place	Yes	32(100)	21(65.62)	-	9(28.12)	2(6.25)
	No	0	-	-		
Conditioning regimen	Myeloablative conditioning	31(96.87)	20(62.5)	-	9(28.12)	2(6.25)
	Non-Myeloablative conditioning	1(3.12)	1(3.12)	-	-	-
Neutropenia duration	> 10 days	28 (87.5)	21(65.62)		7(21.87)	-
	< 10 days	4 (12.5)	-		4(12.5)	-
Outcome	Alive	28 (87.5)	17(53.12)	-	9(28.12)	2(6.25)
	Succumbed	4 (12.5)	4(12.5)	-	-	-

Abbreviations: ALL, acute lymphoid leukemia; CML, chronic myeloid leukemia; CLL, chronic lymphoid leukemia; MM, multiple myeloma; GVHD, graft versus host disease

Overall, IFI occurred in 6.3% of autologous and 13.4% of allogeneic HSCT recipients during the first 3 months after transplantation (Table 2). The infection was diagnosed in 9% of HLA-matched donor transplants, 24% of HLA-mismatched donor transplants, 11.6% of related-donor transplants and 26.3% of unrelated donor transplants. In patients with GVHD (50 cases), an 18% rate of IFI was documented.

When grouped by conditioning regimen, there was a similar incidence of IFI following myeloablative or non-myeloablative conditioning before allogeneic transplantation (9.7% vs. 10% at 3 months, respectively).

As for the underlying hematological malignancies, ALL and then AML were associated with the highest risk (50% of all IFI cases) for IFI occurrence.

Of 32 IFI cases, 28 (87.5%) patients experienced neutropenia for more than 10 days. Accordingly, duration of neutropenia (>10 days, 87.5% vs. <10 days, 12.5%; P-value <0.001) were significantly associated with the incidence of IFI (Table 2).

Aspergillosis was the most prevalent fungal infection being diagnosed in 96.9% of the cases. lung involvement was the most frequent clinical form of the IFI (78.1 %). The three-month attributable mortality among IFI cases was 12.5 % (4 patients).

At the time of IFI, all the patients were receiving antifungal prophylaxis. Twenty-four patients were on fluconazole prophylaxis (75%) while the others had received itraconazole (5 patients) and voriconazole (3 patients).

Median age of the patients who developed IFI was significantly lower compared to the patients without IFI ((Median, IQR: 28.34, 27.44 vs. 38.68, 24.82) (P-value < 0.05)). Mean duration of CVC use was significantly longer in patients who developed IFI compared to those without (36.38 ± 12.52 vs. 24.70 ± 8.17, P-value: <0.001).

Results of the univariate and multivariate analysis of association of demographic and clinical characteristics of patients and development of IFI and study outcomes are shown in Tables 3 to 5. As it can be observed, longer duration of CVC and CMV infection at the time of HSCT were independently associated with increased odds of IFI development (odds ratios of 1.07 and 12.16 respectively; P-value of 0.002 and < 0.001 respectively), while younger age, CMV reactivation following HSCT, and receiving HSCT from an HLA-mismatch donor independently raised the odds of death in patients with IFI (odds ratios of 15.5, 1.07 and 10.70; P-values of 0.005, 0.002 and 0.016 respectively).

**Table 3:** Association of demographic and clinical features with fungal infection development and outcome: Univariate Analyses

Variables	Fungal infection development			Outcome		
	Odds ratio	95% CI	P value	Odds ratio	95% CI	P Value
Age	0.97	(0.95-0.99)	0.004	0.99	(0.99-1.02)	0.574
Sex (female)	1.05	(0.48-2.26)	0.893	1.39	(0.42-4.53)	0.584
Neutropenia (< 10 days)	11.62	(3.97-34.01)	< 0.001	0.10	(0.02-00.45)	0.003
Duration of catheter usage	1.10	(1.06-1.15)	< 0.001	1.04	(1.00-1.08)	0.049
Allogenic HSCT	2.30	(1.07-4.97)	0.032	8.20	(1.84-36.59)	0.006
Unrelated Donor	2.70	(0.85-8.50)	0.089	8.57	(2.50-29.33)	0.001
GVHD	2.45	(1.06-5.67)	0.036	3.34	(1.07-10.43)	0.038
GVHD prophylaxis	2.30	(1.07-4.95)	0.032	8.20	(1.84-36.59)	0.006
Myeloablative conditioning	1.07	(0.13-8.69)	0.945	1.75	(0.17-17.57)	0.631
Non-myeloablative conditioning_ regimen before HSCT	1.03	(0.12-8.44)	0.974	3.00	(0.63-14.35)	0.167
HLA mismatch	3.14	(1.22-8.03)	0.017	3.11	(0.98-9.83)	0.053
Itraconazole	2.54	(1.00-6.45)	0.050	0.67	(0.08-5.39)	0.707
Voriconazole	2.11	(0.66-6.70)	0.204	3.32	(0.85-12.94)	0.083
History of fungal infection	1.32	(0.43-4.04)	0.621	2.60	(0.68-9.83)	0.159
Improvement following previous fungal infection	3.69	(0.34-39.83)	0.282	2.28	(0.18-27.99)	0.518
CMV reactivation	8.34	(3.57-19.46)	< 0.001	8.36	(2.69-25.94)	< 0.001
IVIG	2.14	(0.44-10.36)	0.344	5.68	(1.10-29.22)	0.37
Mucositis	1.52	(0.68-3.41)	0.305	3.27	(0.71-15.07)	0.127
Diabetes	0.47	(0.06-3.66)	0.474	1.20	(0.14-9.68)	0.863
Acute lymphoblastic leukemia	1.98	(0.86-4.55)	0.104	1.94	(0.58-6.41)	0.227
Acute myelogenous leukemia	1.66	(0.67-4.07)	0.269	3.34	(1.07-10.43)	0.038
Hodgkin lymphoma	1.91	(0.73-5.01)	0.184	0.29	(0.03-2.45)	0.261
Non-Hodgkin lymphoma	0.35	(0.04-2.69)	0.314	0.37	(0.04-3.26)	0.377
Myelodysplastic disorder	0.45	(0.02-2.93)	0.484	3.39	(0.38-29.66)	0.269
IV drug abuser	0.55	(0.02-4.15)	0.619	6.00	(0.62-57.52)	0.120
Multiple myeloma	0.12	(0.02-0.53)	0.005	0.25	(0.05-1.16)	0.078
Other disease	1.97	(0.79-4.88)	0.141	1.08	(0.23-5.03)	0.915

**Table 4:** association of demographic and clinical features with fungal infection development: Adjusted Analyses (Multiple Logistic Regression)

Variables	Odds Ratio	95% CI	P Value
Duration of catheter use (days)	1.07	(1.02-1.12)	0.002
CMV reactivation	12.16	(3.44-42.93)	< 0.001

**Table 5:** association of demographic and clinical features with outcome: adjusted analyses (multiple logistic regression)

Variables	Odds Ratio	95% CI	P Value
Age (years)	1.07	(1.02-1.12)	0.002
CMV reactivation	15.50	(2.31-103.91)	0.005
HLA Mismatch Donor	10.70	(1.55-73.83)	0.016

## Discussion

This large scale, single center survey conducted in the main HSCT center of Iran where anti yeast agent administration is the main strategy of antifungal prophylaxis, high rate of IFI post HSCT (9.6%) with definitive predominance of mold agents (97%) were observed. Our single center-large scale study was found as one of the few ones that investigated IFI in HSCT settings in a short period of time. Distinct from previous investigations, we compared the occurrence of IFI in different timelines of HSCT process including pre-engraftment, early post-engraftment and late post-engraftment phase. We did so consider that some patients were taking inpatient antifungal prophylaxis in comparison with those discharged after engraftment while taking outpatient antifungal prophylaxis. Our overall 6-month incidence of 9.4% although is in agreement with previous studies reporting an IFI incidence ranging from 7 to 10%, However, cannot be compared with other studies investigating late or very late IFI in a time when the rate of chronic GVHD as a significant predisposing factor for IFI in HSCT recipients would be differed. In our population, almost 15% developed GHVD.

Both univariate and multivariate analyses exhibited factors other than the type of allogenic HSCT and neutropenia were independently associated with the increased risk of post-transplant IFI.

We found that CMV reactivation significantly raised the odds of IFI development and was associated with undesirable outcomes (odds ratios of 12.16 and 15.4 respectively). This finding is consistent with those of previous observations in HSCT recipients. Multiple factors might contribute to CMV reactivation including immune suppressant therapy and weakened immune system [18, 19]. Besides, the main option for management of CMV infections in HSCT patients is ganciclovir, which is associated with some serious adverse effects, most notably, neutropenia which further put the host at higher risk for IFI [20].

Higher rates of CMV in HSCT patients with IFI requiring mechanical ventilation have been reported earlier [21]. Later studies indicated that CMV may not independently increase the risk of IFI in HSCT patients [3]. Other investigations indicated that CMV infection could significantly and independently increase the risk of IFI and the effect may be more pronounced for late onset IFI development [22]. More recent studies also prove marginally increased risk of late onset IFIs in HSCT patients with CMV reactivation [23].

Results of the current study indicate that CMV increases the risk of IFI, and the effect may be greater than previously assumed, as it can be seen in significantly increased odds where CMV reactivation increased the risk of IFI by as much as 12 times compared to those without it. It should be noticed that patients were followed for 6 months in our study and about 90% of the IFIs was developed in the pre-engraftment period. These findings could add to the findings of previous studies, as they indicated that CMV can mainly result in increased risk of IFI in later stages following HSCT, while our results indicate an increased risk in the early stages following HSCT.

Reactivation of CMV was also significantly related to increased odds of unfavorable outcomes of IFIs. reactivation of CMV has been speculated to be one of the major causes of mortality following HSCT, and death might occur due to multiple clinical syndromes like pneumonia and encephalitis [24]. The most common site of IFI in our study was lungs. CMV infection is also known to cause pneumonia in HSCT patients. Indeed, CMV infection could overlap at sites where IFI is more likely manifested, and this overlapping could lead to dissemination of infection and result in prolonged therapy or death due to IFI. Also, neutropenia is a known risk factor for the worst outcomes in patients with IFI, and it is a known complication of ganciclovir usage [25].

The process of HSCT requires high volumes of intravenous fluid infusion, transfusion of blood and its components and multiple blood sample testing, therefore placement of CVC is necessary. All the patients enrolled in our study had CVC. Our findings show that longer duration of CVC usage is significantly related to IFI development. CVC is a known risk factor for *Candida* bloodstream infections resulting from [25]. The relation between increased duration of CVC usage and IFI development was reported in previous studies as well [26].

The most prevalent type of IFI was *Aspergillus* and mean duration of catheter placement in patients who developed IFI was significantly higher compared to those who didn’t (36.38 vs 24.7 p-value: <0.001). These findings show that CVC may actually be a risk factor for IFIs.

Previous studies have indicated that GVHD is an important risk factor for IFI [4, 27]. Our investigation has also demonstrated that GVHD is a significant risk factor for IFI development and unfavorable outcome. However, multivariate analysis did not document any significant association.

Results of the current study indicate that age might be an independent risk factor for worse outcomes in patients developing IFI following HSCT. older age has been reported as a risk factor for infections in HSCT patients [28]. However, in our study, the mean age of patients who developed IFI was significantly lower compared to those who didn’t (28.43 vs 38.68, p-value: 0.03). This indicates that lower age might be an independent risk factor for worse outcomes from IFI. It may be contemplated that although older age in general may be a risk factor for infection development following HSCT, IFIs might result in worse outcomes in younger patients.

Our results indicated that HLA mismatch status in patients undergoing allogenic HSCT was significantly associated with increased odds of worse outcomes with IFI. Univariate analysis indicated that HLA mismatch status was also associated with increased odds of IFI development, although multivariate analysis did not confirm this relationship.

Previous studies reported that HLA mismatch status has been significantly associated with IFI development [15]. Our study shows that IFIs were more prevalent in patients receiving HSCT from HLA mismatched donors and the outcomes of IFIs in these patients could be significantly worse. This finding may be because of more prevalent development of GVHD and need for more aggressive approaches to manage these complications, also prolonged duration of neutropenia in patients receiving HSCTs from HLA mismatched donors.

Results of the current study failed to show significant relationship between some of the reported risk factors, such as corticosteroid therapy, and development of IFI or worse outcomes from this infection. In HSCT recipients, although multiple risk factors might contribute to IFI development, most of them may not have an independent effect.

Our study had limitations. The most notable result of the current study was the impact of CMV reactivation on IFI development and its outcomes. As mentioned earlier, most of the effects could be caused by the ganciclovir used in the management of CMV disease. However, the effects of ganciclovir therapy were not evaluated in development or outcomes of the IFI in HSCT patients because of lack of a control group. Future studies should focus on the effects of this medication on a larger population of patients.

In summary, the 6-month incidence of post-HSCT IFI was high despite prophylactic use of an azole drug. IFI represents a common complication for allogeneic HSCT recipients in comparison with those receiving autologous HSCT. Considering the definitive predominance of mold IFI (97%) in our population receiving commonly fluconazole prophylaxis, the need for risk-adapted antifungal prophylaxis and changing the local strategy of prophylaxis is posed. Many factors could account for the higher risk and poor prognosis of IFI in HSCT recipients.

## Conclusion

IFIs were found to be relatively common complication of allogenic HSCT and continue to be associated with poor prognosis. CMV infection can be independently associated with IFIs and unfavorable outcomes, while HSCT HLA mismatch donors increase the risk of unfavorable outcomes. As a result, tailoring antifungal prophylaxis in these groups of high-risk hosts is crucial.
